# Some Physiological Factors of the Neuroses of Childhood

**Published:** 1895-12

**Authors:** 


Some Physiological Factors of the Neuroses of Childhood. By B. K. Rochford, M. D., Professor of Physiology and Clinician to Children's Clinic, Medical College of Ohio ; Member of Association of American Physicians and American Pediatric Society, etc. Duodecimo, pp. viii,—122. Price, $1.00. Cincinnati : The Robert Clarke Company. 1895.

This work appeared first as a series of papers in *Archives of Pediatrics*. Dr. Rochford speaks with authority, experiments in the physiologic laboratory being, so far as possible, the basis for his statements. He justly considers the chapter on auto-intoxication the most important in the book. Next in importance should be placed the chapter on reflex irritation. The concluding chapter on excessive nerve activity might profitably be read by all parents and educators. The perusal of the volume will well repay not alone the pediatrist, but the general practitioner as well.

